# Evaluation of apoptosis indexes in currently used oral alpha- blockers in prostate: a pilot study

**DOI:** 10.1590/S1677-5538.IBJU.2017.0668

**Published:** 2018

**Authors:** Mehmet Demir, Yigit Akin, Kubra Asena Kapakin Terim, Mehmet Gulum, Evren Buyukfirat, Halil Ciftci, Ercan Yeni

**Affiliations:** 1 Department of Urology, Harran University School of Medicine, Sanliurfa, Turkey; 2 Department of Urology, Izmir Katip Celebi University School of Medicine, Izmir, Turkey; 3 Department of Pathology, Ataturk University School of Veterinary Medicine, Erzurum, Turkey; 4 Department of Urology, Hacettepe University School of Medicine, Ankara, Turkey; 5 Department of Anaesthesiology, Harran University School of Medicine, Sanliurfa, Turkey

**Keywords:** Prostate, Prostatic Hyperplasia, 5-alpha Reductase Inhibitors

## Abstract

**Objectives:**

Apoptosis effect of oral alpha-blockers is known in the prostate. Apoptosis index of silodosin has not been proved, yet. Aims are to present apoptosis index of silodosin in prostate and to compare this with other currently used alpha-blocker's apoptosis indexes together with their clinical effects.

**Materials and Methods:**

Benign prostatic hyperplasia (BPH) patients were enrolled among those admitted to urology outpatient clinic between June 2014 and June 2015. Study groups were created according to randomly prescribed oral alpha-blocker drugs as silodosin 8mg (Group 1; n=24), tamsulosin 0.4mg (Group 2; n=30), alfuzosin 10mg (Group 3; n=25), doxazosin 8mg (Group 4; n=22), terazosin 5mg (Group 5; n=15). Pa- tients who refused to use any alpha-blocker drug were included into Group 6 as control group (n=16). We investigated apoptosis indexes of the drugs in prostatic tissues that were taken from patient's surgery (transurethral resection of prostate) and/or prostate biopsies. Immunochemical dyeing, light microscope, and Image Processing and Analy- sis in Java were used for evaluations. Statistical significant p was p<0.05.

**Results:**

There were 132 patients with mean follow-up of 4.2±2.1 months. Pathologist researched randomly selected 10 areas in each microscope set. Group 1 showed statisti- cal significant difference apoptosis index in immunochemical TUNEL dyeing and im- age software (p<0.001). Moreover, we determined superior significant development in parameters as uroflowmetry, quality of life scores, and international prostate symptom score in Group 1.

**Conclusions:**

Silodosin has higher apoptosis effect than other alpha-blockers in prostate. Thus, clinic improvement with silodosin was proved by histologic studies. Besides, static factor of BPH may be overcome with creating apoptosis.

## INTRODUCTION

Benign prostatic hyperplasia (BPH) is one of the most frequent diseases in aging men (1). BPH contains static and dynamic factors and these contribute to urinary obstruction during its process (1). Alpha-blocker drugs are initially used as medical treatment of choice (2). Besides, the exact mechanisms of these drugs are still under clinical and laboratory investigations. Nevertheless, selective alpha-receptor blockers are currently used for symptomatic BPH (3). These drugs certainly block-ade alpha-receptors in prostatic tissue. Thus, clinical symptoms of BPH can be reduced. Moreover, these are mostly related with dynamic obstruction of BPH. Previous studies pointed some apoptosis, which is a programmed cell death, in prostate by some of these drugs (4). Additionally, it could not have been proved that the apoptotic action of alpha-blockers could contribute to their clinical efficiency in BPH (5). According to our best knowledge, published studies on apoptotic effect of alpha-blockers did not investigate silodosin and its clinical reflection due to apoptosis.

In the present study, we aimed to compare apoptosis index of currently used oral alpha--blockers in prostate. Furthermore, we evaluated the clinical reflection of apoptotic indexes of silodosin and other alpha-blockers in prostate, as the first in published literature. Our hypothesis was high selectivity of alpha-blockers can lead to more apoptosis in prostate.

## MATERIALS AND METHODS

### Study Design

This study was a retrospective view of prospective collected data and open-labelled and non-randomized clinical investigation. All procedures performed in the present study involving human participants were in accordance with the ethical standards of our institutional research committee and with the 2008 Helsinki declaration and/or its later amendments or comparable ethical standards. Additionally, all patients understood the treatment and aim of the study. The written informed consents were obtained. Ethical committee of our institute approved the study and numbered as “14115”.

Exclusion criteria included the use of 5-alpha reductase inhibitors and/or phytotherapy, presence of prostate cancer, any prostatitis, previous prostate surgery or other minimally invasive in-terventions for prostate, senile dementia, urinary dysfunction such as neurogenic bladder, post voiding residual urine (PVR) >100mL, bladder neck sclerosis, Alzheimer's disease, urethral stricture, bladder stone, urinary tract infection, alpha-blockers drug hypersensitivity, hepatic and/or renal impairment, severe cardiovascular disease and any other cancer. Patients with prostate specific antigen (PSA) >4ng/dL with/without rigid nodule in digital rectal examination (DRE) were excluded for further investigations with prostate biopsy.

BPH patients admitted to urology outpatient clinic between, June 2014 and June 2015, were enrolled. Data was recorded prospectively and was evaluated retrospectively.

### Patient data

Demographic data included age, comorbidities, previous operation history, physical examination including DRE, blood analysis including prostate specific antigen (PSA), liver and kidney functions (creatinine, blood-urea-nitrogen), urinalysis, uroflowmetry (UFM) (Solar Uroflow, Medical Measurement Systems, Inc. Dover, NH 03820, USA) and determination of PVR (The Bio-Con 500, Medline LA, CA 90245, USA), International prostate symptom score (IPSS), Quality of life (QoL) index, transrectal ultrasonography of prostate (TRUS) with 7.5Mhz probe (Sonoline SL 450, Siemens AG, Erlangen, Germany) were performed.

The groups were created according to randomly prescribed oral alpha-blockers: silodosin 8mg (Group 1; n=24), tamsulosin 0.4mg (Group 2; n=30), alfuzosin 10mg (Group 3; n=25), doxazosin 8mg (Group 4; n=22), terazosin 5mg (Group 5; n=15). The control group (Group 6; n=16) consisted of BPH patients who had not used any alpha-blocker/or did not want to use any drug for BPH; transurethral resection of prostate (TURP) was performed. The prostatic tissues were taken from TURP operations and/or prostatic biopsies. The biopsies were performed in patients who were in clinical follow-up and had suspicious prostate nodule in DRE and/or suspicious change in PSA level.

The IPSS and UFM, QoL indexes were recorded before drug administration, and after 1^st^, 6^th^, and 12^th^ month of drug administration.

### Histopathology

The experienced pathologist evaluated all tissues and TUNEL immunochemistry was used for determining apoptosis indexes in prostatic tissues that were removed and immediately half of them were fixed in 10% neutral buffered formalin. Then, dehydration in the graded ethanol series and clearing with xylene, the sample material was placed into paraffin. Immediately after, 4μm-thick sections were stained with Haematoxylin-Eosin (HE).

### Detection of apoptotic cells

The terminal deoxynucleotidyl transferase-mediated dUTP nick end-labelling (TUNEL) was used to determine apoptotic cells by stain using a commercial ready-to-use kit (In Situ Cell Death Detection Kit, POD, Roche, Mannheim, Germany). All steps of the process were performed according to instructions of manufacturer. Shortly, 4μm paraffin sections were prepared on silanized slides. Then, deparaffinization and rehydration were performed and slides were digested with proteinase K (20μg/mL, 30 min.) and quenched with 3% hydrogen peroxide in methanol. The incubation was performed in a humidified chamber in 200μL of TUNEL (TdT and label solution) at 37°C for 60 min. and with POD converter at 37°C for 30 min. The sections were then handled with DAP for 5 min., washed with PBS and counterstained with Mayer's haematoxylin.

Tissue sections were evaluated by high power light microscopic examination Olympus Bx52 with DP72 camera system. All immunohistochemically staining were estimated with an image processing system (Olympus, DP2-BSW). Each TUNEL stain specimen was examined according to 10 randomly selected areas of approximately X40 objectives.

The scores were derived semi-quantitatively using light microscopy on the preparations from each slide and were reported as follows: none: -, mild: +, moderate: ++, severe: +++, and very strong: ++++. Additionally, we used quantification program as Image Processing and Analysis in Java (ImageJ 1.51j8, NIH, USA) for analysing the apoptosis indexes in images. All results were double-checked.

### Statistical analyses

The Statistical Package for the Social Sciences (SPSS) V 16.0 was used for statistical analyses. One-way ANOVA was used for comparing mean data among groups. The significant p was accepted as p<0.05.

## RESULTS

We evaluated 132BPH patients with mean follow-up of 4.2±2.1 months. Demographic data was comparable among groups (Table-1). The mean baseline maximum urine flow rate in uroflowmetry (Qmax) in Group-6 was 11.6±5.1 and we did not include this into statistical analyses. However, the baseline mean Qmax was comparable in all groups when we included data of Group-6 in analyses. The alteration in Qmax is presented in Table-2. There was significant development in Qmax with silodosin (Group-1) during follow-up. This rate was kept during up to 1 year. We did not include Group-6 into Table-2 because we wanted to compare just clinical effects of alpha-blockers.

**Table 1:** Demographic data of groups.

Parameter	Group 1 (n=24)	Group 2 (n=30)	Group 3 (n=25)	Group 4 (n=22)	Group 5 (n=15)	Group 6 (n=16)	P value
**Age (years)**	68.1±8.5	70.7±8.5	68.1±8.4	70.2±8.8	73.3±8.2	67.7±9.5	0.4
**PSA (ng/dL)**	3.2±1.6	2.8±1.7	2.9±1.8	3.1±1.9	2.9±1.7	3.1±2.2	0.9
**Serum creatinine (mg/dL)**	1.4±2.1	1.3±1.6	1.2±1.1	0.9±0.3	1.1±0.6	1.4±0.8	0.5
**Prostate volume (mL)**	37.4±4.7	38.2±3.8	36.1±4.1	35.2±3.7	37.2±4.3	36.1±5.1	0.1

**PSA** = Prostate specific antigen

**Table 2:** Comparison of clinical findings on uroflowmetry in Groups.

Parameter	Group 1 (n=24)	Group 2 (n=30)	Group 3 (n=25)	Group 4 (n=22)	Group 5 (n=15)	P value
**Mean Qmax at baseline**	11.2±3.8	12.4±4.1	12.1±3.5	12.9±3.3	11.8±2.7	0.57
**Mean Qmax at the 1st month**	18.8±8.1	16.2±7.7	14.1±7.2	13.4±4.9	13.5±6.1	0.04*
**Mean Qmax at the 6th month**	18.9±8.8	16±7	14.3±6.8	13.4±4.9	13.2±6.3	0.04*
**Mean Qmax at the 12th month**	18.6±9.2	16.4±8.1	14±5.7	13.1±4.7	13±5.9	0.03*

**Qmax =** Maximum flow rate in uroflowmetry (mL/sec)

* Statistical significant p value.

Additionally, the IPSS scores were significant decreased with silodosin (Group-1) then other groups in 1^st^, 6^th^, and 12^th^ months of follow-up (respectively; 0.04, 0.04, 0.003). The QoL scores were significant developed with Silodosin than other drugs. The most significant difference was obtained in the 12^th^ month of treatment (P=0.003). We did not include Group-6 in comparison of IPSS and QoL since we were comparing the clinical effects of alpha-blockers.

**Figure 1:**
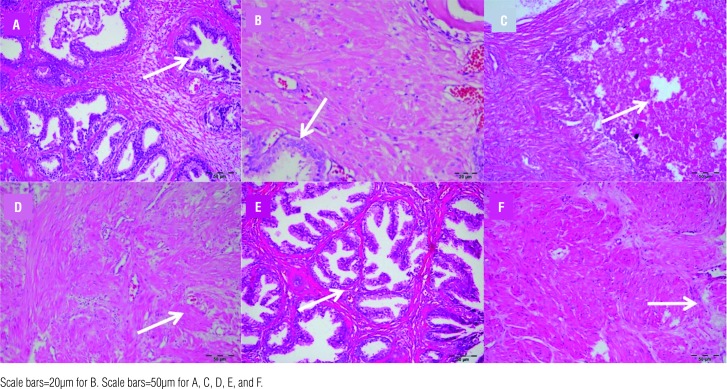
The Haematoxylin-Eosin dyed view of groups. A) The arrow shows glandular reproduction and inflammation in cells of Group-1, B) The more decreased level of inflammation was observed in Group-2 with Haematoxylin-Eosin dying. C) The arrow shows glandular hyperplasia in Group-3, D) The arrow shows glandular hyperplasia with mild amyloid deposition in Group-4, E) The arrow shows glandular hyperplasia with intertubular of lymphocytes and macrophages were creating inflammation in Group-5, F) The most inflammation was observed in Group-6.

In pathology examination, 10 random areas were evaluated in each microscope slide. However, the apoptosis index of Group 3 and 4 was very close to apoptosis index of Group-1. Never-theless, in statistical analyses Group-1 had significant apoptosis index (P<0.001) (Table-3). The HE stained tissues showed some cystic degenerated cells and reproduction of these into tubule lumen. The most inflammation was observed in Group-6 and the lowest level of inflammation was obtained in Group-2 (Figure-1) (Table-3).

**Table 3:** Comparison of apoptosis indexes of Groups.

Parameters	Group 1 (silodosin) n=24	Grouop 2 (Tamsulosin) n=30	Group 3 (Alfuzosin) n=25	Group 4 (Doxazosin) n=22	Group 5 (Terazosin) n=15	Group 6 (Controls) n=16	P Value
Apoptosis index in glandular epithelium	++++ (227**)	++ (161**)	+++ (182**)	+++ (184**)	+++ (188**)	+ (119**)	P<0.001*
Apoptosis index in myocytes	++++	++	+	+++	+++	+	P<0.001*
Inflammatory cells	+++	++	+	+++	+++	+	N.A.
Apoptosis index in vascular endothelial cells	++++	++	+	+++	++	+	P<0.001*
Apoptosis index in stroma	++++	++	++	+++	+++	+	P<0.001*

* Statistical significant P value.

**Figure 2:**
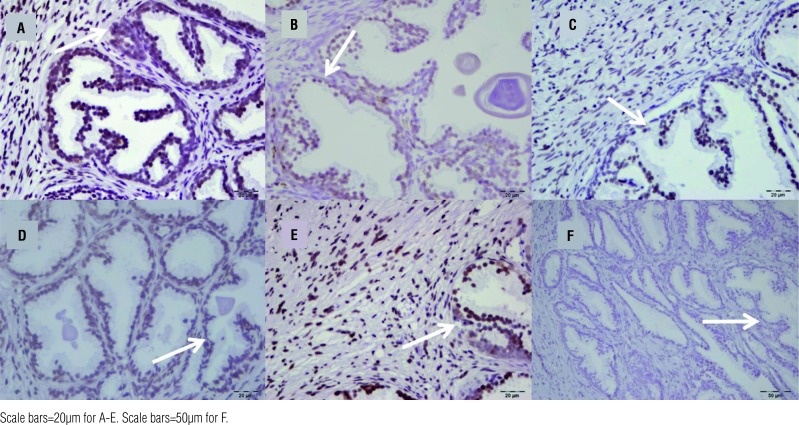
Apoptosis indexes in immunohistochemistry with TUNEL dyeing. A) The most apoptosis was observed in Group-1 (Arrow shows apoptotic cells), B) There was mild apoptosis in Group-2 (Arrow), C) Apoptotic gland in Group-3 (Arrow), D) Apoptotic glands around tissue in Group-4 (Arrow), E) Apoptosis in Group-5 (Arrow), F) The decreased level of apoptosis in Group-6 (Control) (Arrow).

** The numbers were recorded from Image Processing and Analysis in Java software by evaluation of Figure 2.

**N.A** = Not assessed

The immunohistochemically TUNEL staining showed significant differences for apoptosis among Groups (Figure-2). The numbers that were got from Image J Software were also added into Table-3.

The most seen side effect of alpha-blockers was dizziness. However, 3 patients reported anejaculation with silodosin and 1 patient reported this side effect with tamsulosin. No patient withdrawal the treatment.

## DISCUSSION

Apoptosis is the programmed cell death as part of cell's lifecycle (6). It can be induced and can be increased by drugs. The hyperplasia index occurs much more than apoptosis index in BPH. Up till now, apoptotic effects of terazosin (7), Doxazosin (7), alfuzosin (8), and tamsulosin (9) were published in the literature. Increased apoptosis may decrease prostate volume and static factor in BPH can be overcome. Thus, the possible clinical efficacy of these drugs can be beneficial for symptomatic BPH. However, according to our best knowledge, there has not been published any study on clinical effects of these processes (4, 5, 10). Nevertheless, oral alpha-blockers can increase apoptosis in prostatic tissue whether in glands and/or smooth muscles. In the present study, we compared apoptosis indexes of actually used oral alpha-blockers. Additionally, we evaluated clinical effects of apoptotic indexes of these drugs. Silodosin was the most effective alpha-blocker and had the most apoptotic index in prostate. To our best knowledge, this is the first study that apoptosis of silodosin on prostate was showed and was compared with other alpha-blockers. Moreover, clinical effects of apoptosis were presented.

We found significant higher apoptosis index could be obtained with silodosin then other drugs. Moreover, there was an interesting significant clinical reflection in favour of silodosin (11). Yoshida et al. reported that silodosin was more significantly effective then tamsulosin on voiding as well as on uroflowmetric parameters in short-term (12). Our findings were parallel to them. Silodosin provided significant increase in Qmax and decrease in IPSS than other alpha-blockers. We think that this may be related with silodosin's highly selective effect on alfa-1a receptors (11, 12).

Miyakita et al. reported significant improvement of QoL with silodosin (13). We agree with them and there was significant development in QoL with silodosin than other alpha-blockers. We strongly think that this is another reflection of highly selective blockade activity of silodosin on alpha-1A receptors (14). High QoL parameters can be obtained with reduced nocturnal urination, relieved urination, and effective emptying bladder. In our study, reduced nocturia was the most effective developed symptom that could contribute to improve QoL parameters.

On the other hand, Chlosta et al. reported apoptosis with alpha-blockers in prostate and stated that there was no reflection of these on BPH's clinical symptoms (5). We do not agree with them. There were significant clinical improvements with silodosin in terms of decreased IPSS and increased Qmax with developed QoL, in the present study. Creta et al. reported improved urodynamic parameters with silodosin (15). Our findings supported their report. This may be another proof of over coming static factor of BPH by using silodosin. However, there is need of more studies on urodynamic outcomes of silodosin usage.

Partin et al. reported apoptosis with doxazosin mediated transforming factor beta-1 (10). Garcia-Cazare et al. revealed induced mitochondrial p53 translocation by alpha-blockers (6). Un-fortunately, we did not study on molecular mechanism of Silodosin's effectivity. This may be subject of our future study.

There were some side effects related with alpha-blockers. Dizziness was the most seen side effect. It healed in course of therapy spontaneously. It was very well known that alpha blockers can cause dizziness in terms of its pharmacological mechanism (16). However, there was no dizzi-ness with silodosin. Anejaculation was the other most seen side effect in 3 patients using silodosin and in 1 patient using tamsulosin. Anejaculation did not cause withdrawal of silodosin as well as of tamsulosin. The possible mechanism was discussed before (17). On the other hand, Moon et al. concluded safety of Silodosin in a recent study (18). We agree with Moon et al. (18) and the safety the drug was proven in the present study.

We have some limitations in the present study. At first, it was an open labelled non-randomized retrospective pattern study. Low numbers and unbalanced specimen distribution of patients in groups are one of the other limitations. However, we showed apoptosis in prostatic tissue, we did not study on accurate molecular mechanism of alpha-blockers in prostate. Furthermore, we did not detect for prostate volume of patients because some of them underwent TURP. Besides, we did not compare operational data according to used alpha--blockers. Nonetheless, we focused on apoptosis indexes and clinical effects of alpha-blockers. Lastly, Group-6 was not a real control group, however, we included patients suffering from severe lower urinary tract symptoms (LUTS) into that group. At that time, measurement of the obstruction due to LUTS come into question and normally it brings to use urodynamics. We did not perform any urodynamics to LUTS patients in the groups.

The goals of the present study are the highest apoptotic effects of silodosin were showed in histological and immunochemistry staining with its clinical reflection in terms of improved IPSS, QoL, and Qmax. Additionally we focused on effects of si-lodosin in the present study. This study is the first in the literature that clinically analyse silodosin's histologic effects. All these are unique in the literature.

## CONCLUSIONS

Silodosin has the highest levels of apoptosis index in the prostate among currently used alpha-blockers during medical treatment of BPH. Urination symptoms can be reduced and high QoL index can be gained with silodosin. Thus, static factor during BPH can be reduced with silodosin. There is need of more studies with molecular investigations and clinical urodynamic examinations on this issue with high number of patients.
